# Exploring the Regulatory Effect of Hydroxytyrosol on Ovarian Inflammaging Through Autophagy-Targeted Mechanisms: A Bioinformatics Approach

**DOI:** 10.3390/nu17091421

**Published:** 2025-04-23

**Authors:** Xiaoyang An, Xiaoyu Guo, Meng Cai, Meihong Xu

**Affiliations:** 1 Department of Nutrition and Food Hygiene, School of Public Health, Peking University, Beijing 100191, China; 2010306226@stu.pku.edu.cn (X.A.); 2316392165@bjmu.edu.cn (X.G.); 1810306215@pku.edu.cn (M.C.); 2 Beijing Key Laboratory of Toxicological Research and Risk Assessment for Food Safety, Peking University, Beijing 100191, China

**Keywords:** ovarian aging, hydroxytyrosol, non-canonical selective autophagy, inflammaging, network pharmacology, molecular docking, molecular dynamics simulation

## Abstract

**Background/Objectives:** Ovarian aging represents a critically important aspect of female senescence. It not only denotes the loss of fertility but is also accompanied by a series of physiological changes and the aging of other organs. Hydroxytyrosol (HT), a natural polyphenolic phytocompound, has been demonstrated to exhibit remarkable effects in regulating autophagy, inflammation, and the aging process. However, the relationship between HT and ovarian aging, as well as the specific underlying mechanisms, remains poorly understood. **Methods:** In this study, network pharmacology, molecular docking, and molecular dynamics simulation were employed to explore the regulatory effect of HT on ovarian inflammaging via autophagy-targeted mechanisms. **Results:** Through network pharmacology analysis, this study successfully identified 10 hub genes associated with ovarian aging regulation. Notably, four out of the top five hub genes were found to be closely related to autophagy regulatory pathways. Further investigation revealed the pivotal role of ATG7: HT may regulate ovarian inflammaging through activating the FIP200 (focal adhesion kinase family interacting protein of 200 kD)-dependent non-canonical selective autophagy pathway. The results of molecular docking indicated that ATG7 has a strong binding ability with HT. Molecular dynamics simulation further verified the binding stability between the two. **Conclusions:** By analysis, a possible pathway for HT to regulate ovarian inflammaging via non-canonical selective autophagy was explored, providing cues for further research.

## 1. Introduction

Ovarian aging, recognized as a critical hallmark of female aging, is characterized by a rapid decline in reproductive capacity after reaching a certain age threshold, accompanied by the deterioration of multiple systemic and organ functions [1]. According to demographic projections by the World Population Report, the global elderly population (aged 65 years and above) is anticipated to comprise one-third of the total population by 2050, underscoring ovarian aging as a significant health challenge for menopausal women and a major public health concern in the context of global aging [2].

Inflammaging, one of the crucial downstream mechanisms of ovarian aging, is characterized by progressive and sustained systemic pro-inflammatory stress. This age-associated inflammation typically occurs independent of infection, termed sterile inflammation [3]. A hallmark of inflammaging is the increased and persistent elevation of pro-inflammatory cytokines such as interleukin-6 (IL-6), tumor necrosis factor-alpha (TNF-α), and C-reactive protein (CRP), which negatively impact various cellular and tissue functions [4]. These cytokines contribute to immune senescence, metabolic dysregulation, cardiovascular deterioration, and neurodegenerative disorders [5].

Autophagy represents a fundamental cellular response mechanism under stress conditions, and its activity progressively diminishes in the ovary with advancing age, representing a pivotal marker of ovarian aging. Particularly in older women, reduced autophagic activity is frequently accompanied by enhanced granulosa cell apoptosis, increased reactive oxygen species (ROS) production, and elevated cellular mortality rates [6]. Evidence indicates that autophagy significantly regulates inflammaging by suppressing excessive pro-inflammatory cytokine expression in the ovary, thereby mitigating chronic low-grade inflammation [7]. Specifically, autophagy reduces excessive ROS generation, subsequently inhibiting the activation of NLRP3 inflammasome and its pro-inflammatory effects [8]. Moreover, autophagy can attenuate chronic ovarian inflammation by suppressing the overactivation of the NF-κB signaling pathway [9,10].

Autophagy processes are categorized as selective or non-selective. Non-selective autophagy occurs continuously under physiological conditions, independent of specific signals or substrates, randomly degrading cytoplasmic components to maintain cellular homeostasis and basic nutrient supply. Selective autophagy, on the other hand, refers to the process in mammalian cells where specific damaged or excess organelles, protein aggregates, and pathogens are targeted for degradation. This process includes types such as mitophagy and ribophagy, among others, which help maintain cellular health by removing harmful or unnecessary components in a regulated manner [11]. In selective autophagy, most processes rely on microtubule-associated protein 1 light chain 3 (LC3) as an autophagosome marker. LC3 specifically recognizes and binds selective autophagy receptors (SARs) through their LC3-interacting regions (LIRs), facilitating selective substrate recruitment and subsequent degradation—a process termed LC3-dependent selective autophagy. Additionally, a novel non-LC3-dependent selective autophagy pathway has recently been identified. In this process, selective autophagy receptors directly interact with FIP200, recruiting autophagosome formation directly at the cargo site without LC3 involvement. These receptors contain an FIP200-interacting region (FIR), binding specifically to the C-terminal claw domain of FIP200. Notably, FIR sites often overlap with LIR sites, suggesting a competitive and mutually exclusive interaction between FIP200 and LC3 for autophagy receptor binding. Thus, autophagy receptors associated with cargoes may participate in either classical LC3-dependent or non-conventional non-LC3-dependent selective autophagy pathways [12,13,14].

Now we know that autophagy is a process by which cells maintain their health by breaking down and recycling damaged components. LIR and FIR motifs are specialized regions of autophagy receptors that bind to LC3 and FIP200 proteins, respectively, and are involved in the selective degradation of substances during the autophagy process. Previous studies also demonstrate that non-LC3-dependent selective autophagy possesses specific roles in counteracting TNF cytotoxicity [15]. Given that TNF is a prominent pro-inflammatory cytokine orchestrating inflammatory responses through gene activation, cell survival, and cell death, regulating autophagic activity represents a promising strategy for attenuating ovarian inflammaging [15].

In recent years, food-derived natural compounds, especially from plant origins, have attracted considerable interest due to their high safety profile and potential nutritional and medicinal values, notably in ovarian aging research. As an organ highly sensitive to aging, ovarian functional decline often precedes deterioration in other systems, influencing female fertility and healthy longevity. Plant compounds exert multiple beneficial effects through antioxidative, anti-inflammatory, anti-apoptotic, and autophagy-regulating mechanisms, providing novel solutions for delaying ovarian aging. HT, a natural polyphenolic compound abundant in olive oil and its derivatives, is particularly advantageous due to its higher oral bioavailability, prolonged half-life, and excellent safety profile compared to other plant compounds [16,17,18]. Recent evidence highlights HT’s significant anti-inflammatory and anti-aging roles by inhibiting inflammatory signaling pathways and activating autophagy-related pathways [19,20,21,22,23,24,25,26]. However, the specific role of HT in ovarian inflammaging and its ability to modulate inflammation via autophagy mechanisms remain unclear and necessitate further investigation.

Utilizing network pharmacology, molecular docking, and molecular dynamics simulation methods, this study explored the potential of HT in delaying ovarian inflammaging. Additionally, it proposed a novel pathway based on non-LC3-dependent selective autophagy to elucidate the mechanism by which HT regulates ovarian inflammaging. Therefore, this study provides new research directions and insights into the nutritional interventions targeting ovarian aging.

## 2. Materials and Methods

### 2.1. Ovarian Aging Data Acquisition and Differential Analysis

The gene expression dataset GSE62093 associated with ovarian aging was acquired from the Gene Expression Omnibus (GEO) database (https://www.ncbi.nlm.nih.gov/geo/, accessed on 14 January 2025). This dataset contains ovarian granulosa cell samples from six young individuals (aged 24–28 years) and six aging individuals (aged 38–42 years), generated using the GPL11154 (Illumina HiSeq 2000) platform. Differential gene expression analysis was performed using the limma package (v3.62.2) in R software (v4.4.2), with significance thresholds set at FDR < 0.2, absolute logFC > 1, and *p* < 0.05. Volcano plots and heatmaps were generated using the ggplot2 package (v3.5.1) and the pheatmap package (v1.0.12).

### 2.2. HT Target Prediction and Autophagy-Related Gene Databases

Potential structural targets of HT were predicted using the following databases: SwissTargetPrediction (http://swisstargetprediction.ch/, accessed on 12 January 2025) [27], PharmMapper (https://lilab-ecust.cn/pharmmapper/, accessed on 12 January 2025), SuperPred (https://prediction.charite.de/, accessed on 12 January 2025) [28], BindingDB (https://www.bindingdb.org, accessed on 12 January 2025) [29], STITCH (http://stitch.embl.de/, accessed on 12 January 2025), Comparative Toxicogenomics Database (CTD; http://ctdbase.org/, accessed on 12 January 2025) [30], Drug-Gene Interaction database (DGIdb; http://www.dgidb.org/, accessed on 12 January 2025) [31], and Traditional Chinese Medicine Systems Pharmacology Database (TCMSP; http://www.tcmsp-e.com, accessed on 12 January 2025) [32]. Autophagy-related genes were obtained from the Human Autophagy Database (HADb, http://www.autophagy.lu/, accessed on 12 January 2025), Autophagy Database (http://autophagy.info/, accessed on 12 January 2025), Human Autophagy Moderator Database (HAMdb, http://hamdb.scbdd.com/, accessed on 12 January 2025) [33], and Molecular Signatures Database (MSigDB, http://www.gsea-msigdb.org/gsea/msigdb/, accessed on 12 January 2025). Gene names were standardized and missing genes complemented by connecting the Ensembl database (http://www.ensembl.org, accessed on 12 January 2025) through the biomaRt package (v2.62.0) under the org.Hs.eg.db package (v3.20.0). The intersections of gene sets were identified using the intersect function in R and visualized using the VennDiagram package (v1.7.3).

### 2.3. KEGG and GO Enrichment Analyses

KEGG and GO enrichment analyses for DEGs were conducted using the clusterProfiler package (v4.14.4) under the org.Hs.eg.db environment (v3.20.0), with *p*-value cutoff set at 0.05. The top 10 categories from GO enrichment were visualized.

### 2.4. PPI Network Construction and Hub Gene Screening

A protein-protein interaction (PPI) network was constructed using the Search Tool for the Retrieval of Interacting Genes/Proteins (STRING, http://string-db.org/, accessed on 21 March 2025) with a confidence threshold ≥ 0.04. Non-interacting nodes were removed. The network was visualized using Cytoscape software (version 3.10.1). Four algorithms (MCC, MNC, Degree, and Closeness) from the CytoHubba plugin were used to rank genes, and the top ten genes with integrated scores from these algorithms were identified as hub genes.

### 2.5. Annotation of Hub Genes Using AutophagyNet and Prediction of LIR and FIR Motifs

Hub genes were annotated by mapping them to the AutophagyNet database (http://www.autophagynet.org/, accessed on 26 December 2024) and SignaLink 3.0 (http://signalink.org/, accessed on 26 December 2024) to explore autophagic functions and pathways related to selective autophagy-related genes [34,35]. The LIR motifs were predicted using the iLIR database (https://ilir.warwick.ac.uk/, accessed on 26 December 2024). The FIR motif consensus sequence (ψΘxxΓ, where ψ represents acidic Asp, Glu, or phosphorylated Ser/Thr residues; Θ indicates hydrophobic Ile, Leu, Val, or aromatic Phe, Tyr, Trp residues; Γ represents hydrophobic Leu, Ile, or Val residues; and x indicates any residue) was identified by comparing sequences from UniProt (http://www.uniprot.org/, accessed on 12 January 2025) [14]. Sequence alignment and motif identification were performed using R software.

### 2.6. Molecular Docking and Molecular Dynamics Simulation

Molecular docking is a computational method used to simulate and predict the binding modes between molecules, such as proteins and small molecules, helping us understand how they interact with each other. Molecular dynamics simulations, on the other hand, are used to simulate the movement and changes of these molecules over time to further understand their binding stability.

The SMILES and SDF structures of HT were retrieved from PubChem (https://pubchem.ncbi.nlm.nih.gov/, 12 January 2025), and the predicted structure of ATG7 was obtained from AlphaFold Protein Structure Database (https://alphafold.com/, accessed on 12 January 2025) [36,37]. Molecular docking was conducted using Discovery Studio 2019 software, calculating LibDock scores and visualizing binding sites. Docking parameters included a center at (x = 26.145, y = 0.632277, z = −20.1966) with dimensions of 20 on each axis. Protein-ligand complexes were prepared with MGLTools software (v1.5.7) in PDBQT format, and binding energies were calculated using AutoDock Vina (v1.2.5) to validate the predicted interactions.

Molecular dynamics simulations were performed using GROMACS software (version 2020.6_AVX2_CUDA_win64). The complex was placed in the center of a cubic box with SPC water model solvent and neutralized by adding four sodium ions. Energy minimization employed the Steepest Descent algorithm, followed by temperature (NVT) equilibration using the V-rescale method (tau_t = 0.1, ref_t = 300 K) and pressure (NPT) equilibration using Parrinello-Rahman (tau_p = 2.0). Simulations were run for 50 ns, with data analysis and visualization performed using Python 3.8.

## 3. Results

### 3.1. Differential Expressed Genes Analysis

**Figure 1:**
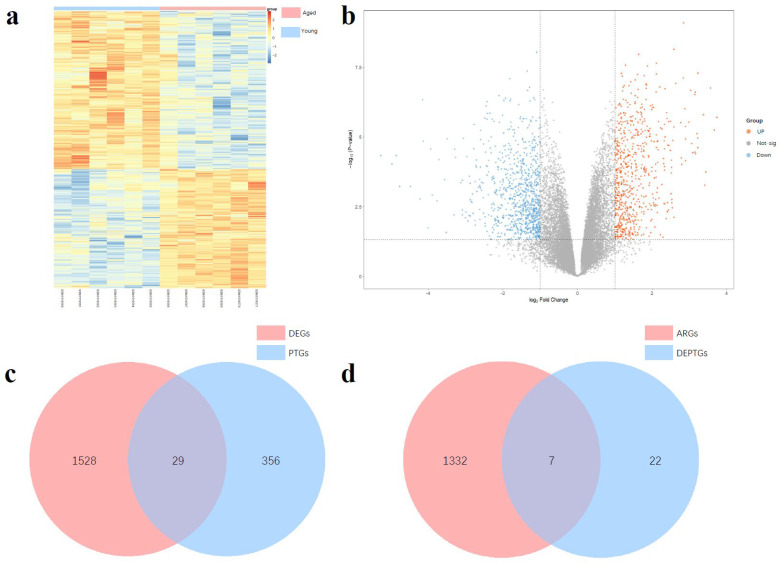
(**a**) Heat map of GSE62093’s differential expressed genes. (**b**) Volcanic map of GSE62093’s differential expressed genes. (**c**) Venn map between DEGs and PTGs, containing 29 DEPTGs of HT. (**d**) Venn map between DEPTGs and ARGs, containing seven AR-DEPTGs.

We selected the GSE62093 dataset from the GEO database for the analysis of differentially expressed genes (DEGs) related to ovarian aging. Following analysis, a total of 1557 DEGs were identified, including 887 downregulated genes and 670 upregulated genes. The DEGs were visualized using heatmaps and volcano plots (Figure 1a,b). Subsequently, we predicted potential structural targets of HT using eight databases: SwissTargetPrediction, PharmMapper, SuperPred, BindingDB, STITCH, CTD, DGIdb, and TCMSP, resulting in a total of 385 predicted target genes (PTGs). The intersection of PTGs and DEGs was then determined, yielding 29 differentially expressed potential target genes (DEPTGs) of HT (Figure 1c). These genes may play critical roles in the mechanism by which HT exerts its protective effects against ovarian aging, providing a basis for further experimental validation.

### 3.2. Screening of Autophagy-Related DEPTGs

We collected a total of 1340 autophagy-related genes (ARGs) from four autophagy-related gene databases: the Human Autophagy Database, Autophagy Database, Human Autophagy Moderator Database, and Molecular Signatures Database. These databases include all three major types of autophagy as well as other relevant genes. By intersecting the ARGs with the DEPTGs, we identified seven autophagy-related differentially expressed HT potential target genes (AR-DEPTGs) (Figure 1d). This process aims to preliminarily explore the role of autophagy in the effects of HT on ovarian aging and to validate the possibility that HT may influence ovarian aging through autophagy.

### 3.3. KEGG and GO Enrichment Analysis

**Figure 2:**
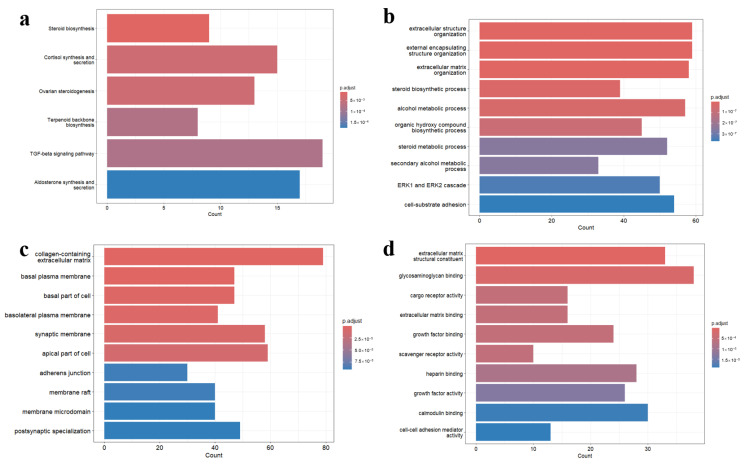
(**a**) KEGG analysis of the DEGs. (**b**) The GO analysis of DEGs (biological processes). (**c**) The GO analysis of DEGs (cellular components). (**d**) The GO analysis of DEGs (molecular functions).

We performed KEGG and GO enrichment analyses on the DEGs, aiming to explore their potential pathways and biological functions (Figure 2a–d). The GO analysis encompassed three categories: biological processes, cellular components, and molecular functions. In the KEGG analysis, the TGF-β signaling pathway was identified as a classical immune regulatory pathway that can either promote inflammatory molecule expression or suppress inflammatory responses. Additionally, this pathway can activate SMAD-dependent and SMAD-independent pathways to induce autophagy. Moreover, the cortisol synthesis and secretion pathway was highlighted, where cortisol, a potent anti-inflammatory hormone, may be associated with age-related chronic inflammatory stress. Within the biological process category of GO analysis, the ERK/MAPK pathway plays a central role in amplifying inflammatory signaling and regulating autophagy initiation. Furthermore, extracellular matrix (ECM) remodeling, commonly observed in chronic inflammatory microenvironments, may influence autophagic substrate clearance. In the molecular function category, growth factor binding was identified, indicating that various pro- inflammatory and anti-inflammatory growth factors act through this function. Notably, some growth factors, such as TGF-β, can induce autophagy, consistent with the KEGG pathway analysis.

### 3.4. PPI Network and Hub Gene Results

**Figure 3:**
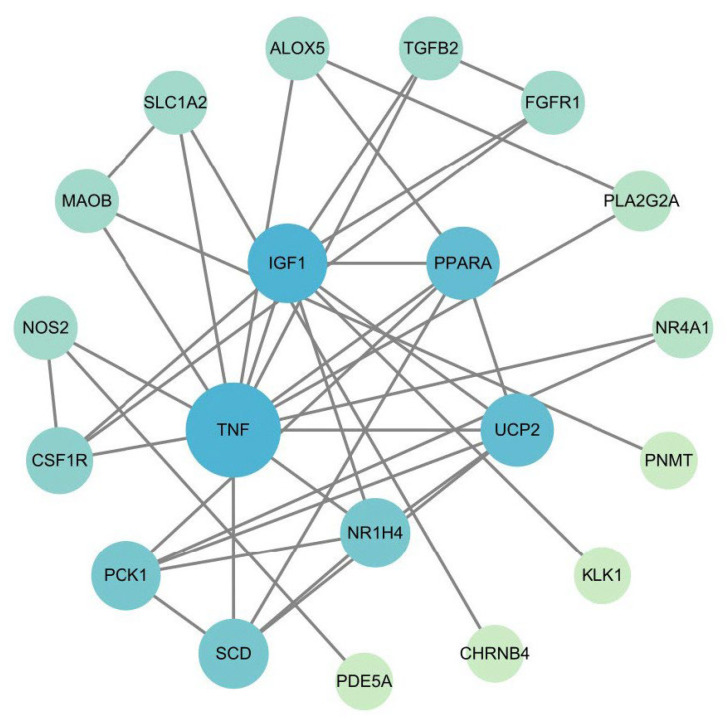
PPI analysis was conducted on the 29 DEPTGs using the STRING database and visualized in Cytoscape (v3.10.1). Critical value confidence score ≥ 0.04 and nodes that do not interact with other proteins are removed from the network. The darker the color, the higher the score of the gene in the PPI network.

We constructed a PPI network for the 29 DEPTGs using the STRING database (Figure 3). Nodes that did not interact with other proteins were excluded from the network. Subsequently, we employed four algorithms provided by the CytoHubba plugin (MCC, MNC, Degree, and Closeness) to evaluate and rank the genes within the PPI network. The top 10 genes identified by combining the results of these four algorithms were selected as hub genes for further analysis. Furthermore, we compared seven AR-DEPTGs with the identified hub genes and found that four autophagy-related genes (IGF1, SCD, UCP2, and TNF) were among the top five hub genes, all ranked highly (Table 1), indicating the important role of autophagy in the HT-ovarian aging process, which warrants further investigation. At last, this integrated analysis of enrichment analysis, autophagy-related databases, and PPI network construction reveals potential links between autophagy and inflammaging during ovarian aging. Additionally, it highlights HT’s potential targets, providing a foundation for subsequent mechanistic investigations.

### 3.5. Functional Annotation Using AutophagyNet and Prediction of LIR and FIR Motifs

To further elucidate the functional relevance of the identified hub genes, we utilized the AutophagyNet database to annotate them, with a particular focus on their directly regulated selective autophagy-related genes, primary cellular localization, and associated signaling pathways (Table 1). The identified SARs were then mapped to the iLIR database to predict whether the corresponding proteins contain potential LIR motifs. Additionally, we compared their sequences with the conserved core sequences of FIR motifs reported in the literature to identify proteins potentially involved in non-canonical selective autophagy pathways (FIR-dependent). Based on these predictions, the SARs regulated by hub genes were classified into three categories:

1. SARs containing only LIR motifs (indicating involvement solely in LC3-dependent selective autophagy);
2. SARs containing only FIR motifs (indicating involvement solely in non-canonical selective autophagy (FIP200 dependent));
3. SARs containing both LIR and FIR motifs (indicating potential participation in both LC3-dependent and non-canonical selective autophagy (FIP200 dependent)).

Following LIR and FIR motif prediction, the SARs were classified as follows: MAP1LC3B, ATG7, and BCL2 contain only FIR motifs, while RB1CC1 contains both LIR and FIR motifs. MAP1LC3B and RB1CC1 were excluded from further analysis because MAP1LC3B encodes LC3B, a core component of LC3-dependent autophagy, and RB1CC1 encodes FIP200, a core component of non-canonical selective autophagy (FIP200 dependent).

### 3.6. Molecular Docking

**Figure 4:**
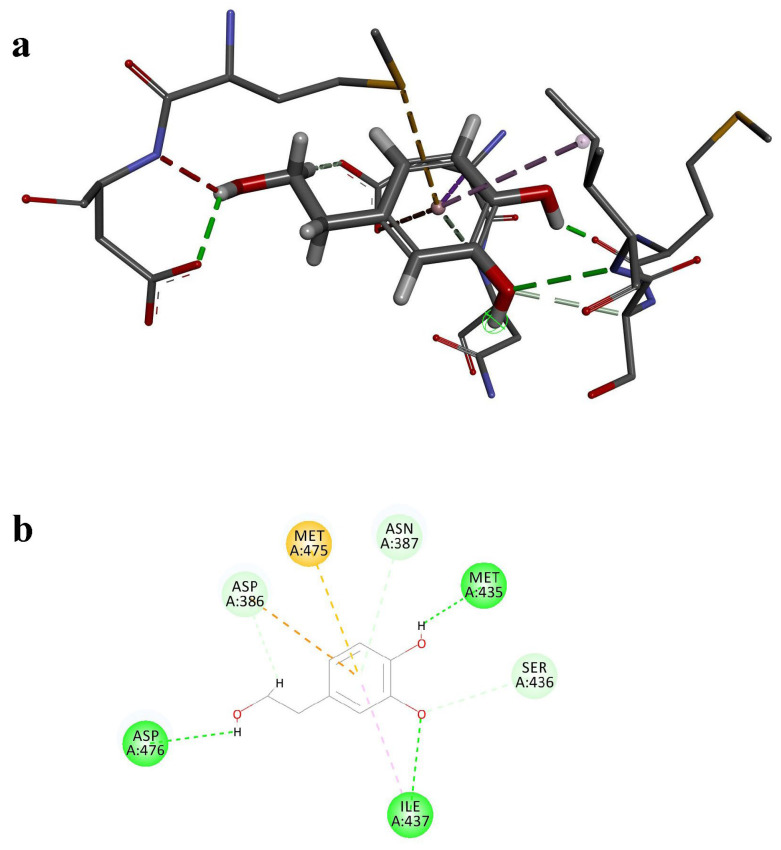
(**a**) The results of the molecular docking ball-and-stick model between ATG7 and HT. The dashed lines represent the various interactions between HT and the protein. (**b**) The visualization of molecular docking sites between ATG7 and HT; the green dashed line in the figure is the conventional hydrogen bonds in the docking results.

Among the SARs related to the hub genes, ATG7 and BCL2 were identified as containing only FIR motifs, suggesting that non-canonical selective autophagy (FIP200 dependent) may play a role in this regulatory process. To explore potential interactions between ATG7 and the small-molecule plant compound HT, we performed molecular docking. The SMILES and 3D SDF structures of HT were obtained from PubChem, while the most probable protein structure of ATG7 was retrieved from the AlphaFold AI prediction database. Molecular docking was conducted using Discovery Studio 2019 to calculate the LibDock score and visualize the ball-and-stick model and binding site (Figure 4a,b). Additionally, AutoDock Vina was employed to calculate the binding energy. The docking results revealed that the LibDock score between ATG7 and HT was 77.92, with a binding energy of −5.785 kcal/mol. Four conventional hydrogen bonds were formed, indicating that the interaction between HT and ATG7 is meaningful and may involve significant biological processes.

Previous studies have demonstrated that the dissociation constant (Kd) of the interaction between ATG7 and ATG3 is approximately 0.35 μM, corresponding to a binding energy of around −8.81 kcal/mol, indicating strong binding affinity between these two proteins [38]. In comparison, the molecular docking results showed that the binding energy between HT and ATG7 was −5.785 kcal/mol, which, although weaker than that of ATG3, still suggests a considerable binding potential. Therefore, the interaction between HT and ATG7 warrants further investigation and validation to elucidate its binding capacity and potential biological functions.

### 3.7. Molecular Dynamics Simulation

To further validate the binding stability of HT and ATG7, a 50 ns molecular dynamics (MD) simulation of the ATG7-HT complex was conducted using GROMACS. The binding stability was evaluated using various parameters including RMSD, RMSF, Rg, SASA, hydrogen bond count, and total energy.

**Figure 5:**
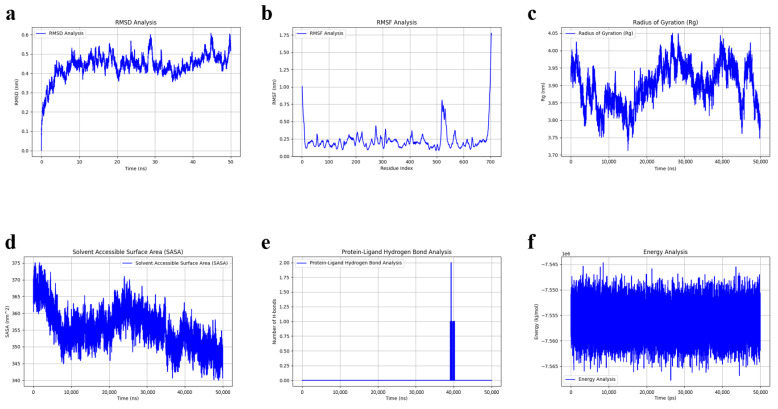
(**a**) RMSD analysis’s result of molecular dynamics simulation. (**b**) RMSF analysis’s result of molecular dynamics simulation. (**c**) Radius of gyration’s result of molecular dynamics simulation. (**d**) Solvent accessible surface area’s result of molecular dynamics simulation. (**e**) Hydrogen bond count’s result of molecular dynamics simulation. (**f**) Total energy analysis’s result of molecular dynamics simulation.

RMSD (Root Mean Square Deviation): The RMSD data indicate that the ATG7-HT complex reached equilibrium after approximately 5 ns, with minor fluctuations around 0.45 nm. This suggests that the complex formed during the simulation is structurally stable, reflecting the behavior we would expect in a biological environment where the interaction between HT and ATG7 is maintained over time. The stability observed here is crucial for the physiological relevance of the complex, indicating that the binding affinity between HT and ATG7 is robust and could potentially be maintained in biological systems (Figure 5a).

RMSF (Root Mean Square Fluctuation): Most residues exhibited RMSF values around 0.25 nm. A low peak was observed between residues 380 and 480, corresponding to the predicted binding site, suggesting a relatively stable interaction between the ligand and protein. Higher RMSF values around residue 530 may represent a flexible region of the ATG7-HT complex (Figure 5b).

Rg (Radius of Gyration): The Rg values remained relatively stable between 3.8 and 4.0 nm, reinforcing that the ATG7 protein maintains its compact structure despite minor adjustments due to HT binding. This behavior reflects a native-like structural stability in a biological environment, suggesting that the HT binding does not cause significant conformational distortions of ATG7 that would disrupt its function (Figure 5c).

SASA (Solvent Accessible Surface Area): The observed decrease in SASA indicates that the ligand (HT) binding leads to a reduction in the surface area exposed to the solvent, suggesting that the binding enhances the stability of the complex by burying hydrophobic regions of the protein. This type of binding-induced conformational change is commonly observed in protein-ligand interactions, supporting the hypothesis that HT binding to ATG7 may modulate its activity in a manner that is consistent with biological processes (Figure 5d).

Hydrogen Bond Count: The number of hydrogen bonds between the protein and ligand remained mostly stable around zero, indicating stable binding throughout the simulation (Figure 5e).

Total Energy: The total energy remaining stable at around −7.55 kcal/mol, which is consistent with a thermodynamically favorable binding between HT and ATG7, implying that the interaction could persist in a cellular environment (Figure 5f).

Together, these results suggest that the HT-ATG7 interaction is not only stable but also reflects a biologically plausible mechanism through which HT may regulate autophagy-related pathways, contributing to the anti-aging effects observed in ovarian aging and inflammaging. The MD simulations provide supporting evidence that HT may act as a potential modulator of ATG7 function, offering a promising mechanism by which HT could influence cellular homeostasis and inflammation in aging.

## 4. Discussion

Ovarian aging, as the pacemaker of female aging, holds significant importance. Currently available interventions include pharmaceutical approaches, which often have safety concerns, and nutritional supplements, which are relatively safer but typically target only a single pathway with limited efficacy. HT, a plant-derived polyphenol with numerous advantages, presents a promising area of study for its role and mechanisms in ovarian aging. Investigating its potential targets may inform targeted experimental designs and address limitations associated with current supplements.

In the current study, we comprehensively explored the potential regulatory effect of HT on ovarian inflammaging via autophagy-targeted mechanisms using integrative bioinformatics and molecular modeling approaches. Our analysis identified 1557 DEGs associated with ovarian aging, of which 29 overlapped with predicted targets of HT (DEPTGs). Among these, seven AR-DEPTGs were identified, suggesting a potential mechanism involving autophagy pathways in the beneficial effects of HT against ovarian inflammaging.

Our KEGG and GO enrichment analyses indicated significant enrichment in pathways such as the TGF-β signaling and cortisol synthesis pathways, as well as biological processes involving the ERK/MAPK pathway and extracellular matrix remodeling. These pathways have been previously implicated in the modulation of inflammation and autophagy during aging [39,40,41,42,43]. For instance, the TGF-β pathway is known to play a dual role, either promoting inflammation through SMAD-independent signaling or suppressing inflammatory responses via SMAD -dependent mechanisms, ultimately influencing autophagy activation [41,42]. Similarly, cortisol pathways have been correlated with chronic inflammatory states associated with aging, reflecting a potential systemic regulatory mechanism of HT [43]. Within the biological process category of GO analysis, the ERK/MAPK pathway plays a central role in amplifying inflammatory signaling and regulating autophagy initiation. In inflammation, the ERK/MAPK pathway activates transcription factors such as NF-κB and AP-1, promoting the expression of pro-inflammatory cytokines like TNF-α, IL-6, and IL-1β [44,45,46]. It also may enhance immune cell survival by activating Bcl-2 family proteins, offering protection in both acute and chronic inflammation [47,48]. However, excessive activation of ERK/MAPK may contribute to immune evasion or autoimmune responses [49]. Regarding autophagy, ERK regulates the expression of autophagy-related genes to enhance autophagic activity in response to stress [50].

Further, the construction of the PPI network and identification of hub genes revealed that IGF1, SCD, UCP2, and TNF were key autophagy-related targets potentially regulated by HT. Specifically, TNF, a pro-inflammatory cytokine, has been extensively linked to inflammaging and autophagy disruption [15]. The identification of IGF1 aligns with its possible role in aging and ovarian function, previously reported to influence autophagy-related pathways [51,52]. SCD and UCP2 involvement further reinforces the complex interplay between metabolic regulation, inflammation, and autophagy processes during ovarian aging [53,54,55,56]. Therefore, our findings suggest that HT may simultaneously modulate inflammation, metabolism, and autophagy to exert its protective effects.

Our molecular docking and MD simulations provided additional support for ATG7 as a plausible direct target of HT. Although the binding affinity between HT and ATG7 (binding energy −5.785 kcal/mol) was weaker compared to the natural protein-protein interactions (e.g., ATG7-ATG3 complex), the sustained stability throughout the MD simulations indicated a meaningful interaction. Previous studies highlight ATG7’s crucial role in autophagy initiation and regulation, further suggesting a mechanistic link through which HT may mitigate ovarian inflammaging. Notably, our analysis of LIR and FIR motifs revealed that ATG7 may participate in non-canonical selective autophagy (FIP200 dependent), indicating a novel pathway of HT-mediated autophagy regulation distinct from classical LC3-dependent pathways.

Our findings contribute to a broader understanding of how dietary polyphenols, such as HT, could nutritionally modulate autophagy and inflammation to mitigate ovarian aging. However, limitations exist, notably the absence of experimental validation of the predicted interactions and pathways. While the bioinformatics and molecular modeling approaches provide valuable insights into the potential mechanisms by which HT modulates ovarian inflammaging, the predictions made in this study are based on computational models that require experimental validation. In response to this limitation, several related in vitro studies have explored the interactions of HT with various biomolecules, providing preliminary evidence supporting our computational predictions. HT has been shown to bind to bovine serum albumin (BSA) through hydrophobic interactions, confirming stable ligand-protein binding similar to the HT-ATG7 interaction predicted in our model [57]. Additionally, HT interacts with soy protein isolate (SPI), modulating its structure and function, suggesting HT’s role in protein regulation relevant to autophagy [58]. Furthermore, HT inhibits the formation of advanced glycation end products (AGEs) in BSA glycation models, highlighting its potential to influence protein modifications, which are important in aging and metabolic diseases [59]. These studies offer valuable insights and experimental strategies, reinforcing that HT interacts with proteins and can modulate cellular processes like autophagy. While they do not directly validate the HT-ATG7 interaction, they support the biological plausibility of HT binding to target proteins.

To substantiate our simulations, future research should include in vitro and in vivo experiments, such as co-immunoprecipitation (Co-IP), fluorescence resonance energy transfer (FRET), or surface plasmon resonance (SPR), to confirm the HT-ATG7 interaction. Additionally, exploring HT’s effects on autophagic flux in relevant cell types and assessing its clinical relevance in terms of dosage, bioavailability, and individual response, will be critical for determining its therapeutic potential. Moreover, HT may also influence other autophagic pathways, such as mitophagy, providing a broader understanding of its role in ovarian aging.

In conclusion, our study provides robust bioinformatics and computational evidence supporting HT’s potential regulatory role in ovarian aging via autophagy-targeted mechanisms. By identifying key molecular targets and pathways, this research sets the foundation for further mechanistic studies and clinical investigations, underscoring the promising role of HT as a natural dietary intervention against ovarian inflammaging.

## 5. Conclusions

This study reveals the potential of HT in alleviating ovarian inflammaging through autophagy-related pathways. By identifying key target genes and regulatory networks, our findings provide new insights into the multi-targeted effects of HT in ovarian aging. These results suggest that HT may serve as a promising nutritional intervention for delaying ovarian senescence, offering a novel strategy to support female reproductive health and healthy aging.

## Figures and Tables

**Table 1:** Top 10 hub genes and their direct regulators from AutophagyNet.

Hub Genes	Direct Regulators
TNF *	BCL2
IGF1 *	No result
UCP2 *	No result
PPARA	BCL2
SCD *	MAP1LC3B ATG7 RB1CC1
NR1H4	No result
CSF1R	No result
PCK1	SQSTM1
TGFB2	RB1CC1
ALOX5	No result

* AR-DEPTGs that were among the hub genes. The genes in the table are sorted from high to low according to their importance.

## Data Availability

Data are contained within the article.

## Abbreviations

The following abbreviations are used in this manuscript:

AR-DEPTGs	autophagy-related differentially expressed potential target genes
ARGs	autophagy-related gene
CRP	C-reactive protein
CTD	Comparative Toxicogenomics Database
DEGs	differentially expressed genes
DEPTGs	differentially expressed potential target genes
DGIdb	Drug-Gene Interaction Database
ERK/MAPK	extracellular signal-regulated kinase/ mitogen-activated protein kinase
FDR	false discovery rate
FIP200	focal adhesion kinase family interacting protein of 200 kD
FIR	FIP200-interacting region
GEO	Gene Expression Omnibus
GO	Gene Ontology
HADb	Human Autophagy Database
HT	hydroxytyrosol
IL-6	interleukin-6
KEGG	Kyoto Encyclopedia of Genes and Genomes
LC3	microtubule-associated protein 1 light chain 3
LIR	LC3-interacting region
logFC	log fold change
MD	molecular dynamics
NF-κB	nuclear factor kappa-light-chain-enhancer of activated B cells
NLRP3	NOD-like receptor family pyrin domain containing 3
NPT	constant pressure and temperature ensemble
NVT	constant volume and temperature ensemble
PPI	protein-protein interaction
PTGs	predicted target genes
Rg	radius of gyration
RMSD	root mean square deviation
RMSF	root mean square fluctuation
ROS	reactive oxygen species
SARs	selective autophagy receptors
SASA	solvent accessible surface area
TCMSP	Traditional Chinese Medicine Systems Pharmacology Database
TGF-β	transforming growth factor-beta
TNF-α	tumor necrosis factor-alpha
